# Donepezil-induced bradycardia in a schizophrenic patient with comorbid neurocognitive disorder: a case report and review of the literature

**DOI:** 10.1186/s13256-024-04454-x

**Published:** 2024-03-27

**Authors:** Nkolika Odenigbo, Stanley Nkemjika, Ayodele Atolagbe, Christian Nwabueze, Connie Olwit, Jeffery Lawrence, Tolulope Olupona

**Affiliations:** 1 Department of Psychiatry, Interfaith Medical Center, Brooklyn, NY USA; 2 Department of Nursing, Makere University, Kampala, Uganda

**Keywords:** Anticholinesterase inhibitor, Donepezil, Schizophrenia, Bradycardia

## Abstract

**Background:**

Trials of cholinergic and glutamatergic agents have improved cognition and memory for the geriatric schizophrenic population. Donepezil is an acetylcholinesterase inhibitor that improves cognition by preventing postsynaptic degradation of hippocampal acetylcholine in patients with mild-to-moderate dementia. Donepezil has been attributed to some adverse effects, especially gastrointestinal symptoms. However, cardiovascular adverse effects are not common as there remains a dearth of literature regarding donepezil-induced bradycardia.

**Case report:**

Hence, we present the case of a 70-year-old Hispanic female with past psychiatry history of schizophrenia who developed bradycardia and syncope following the commencement of low-dose donepezil in the inpatient unit and subsequent resolution with cessation. She had no prior cardiovascular symptoms or diagnosis.

**Discussion:**

Considering there is no baseline cardiac monitoring requirement guideline for patients on Donepezil treatment, pre-assessment electrocardiogram is advised before the commencement of acetylcholinesterase inhibitors. Finally, routine monitoring of vital signs for at least the first 72 hours following the start of donepezil might be good proactive practice for all psychiatrists. Extending this practice to inpatient and outpatient service settings will be worthwhile.

## Introduction

Acetylcholinesterase inhibitor (AI) is a class of medications that improves cognition by preventing post-synaptic degradation of hippocampal acetylcholine in patients with mild-to-moderate dementia [1]. AI has also been described in the literature as an adjunct agent in managing mood symptoms. Donepezil, the most common class of AI, is widely used as the mainstay treatment of mild-to-moderate Alzheimer’s disease (AD). Donepezil’s pharmacodynamics promotes acetylcholine binding to nicotinic acetylcholine receptors in the brain [2]. Hence, improving the ability to interact with people, memory function, and attention. As with most pharmacological agents, donepezil trials in randomized controlled trial studies described myriads of documented attributable adverse effects, which are cholinergic dependent [3, 4]. Notable documented adverse effects are gastrointestinal symptoms, while other side effects are sparingly reported in the literature [4]. Cardiovascular adverse effects are uncommon, but they have been mentioned in hospital settings as syncope, bradycardia, atrioventricular block, and QT prolongation among patients with comorbid cardiac problems [5]. However, there remains a paucity of literature regarding donepezil-induced bradycardia (DIB) in patients without comorbid cardiovascular disease. Bradycardia owing solely to donepezil pharmacotherapy without concurrent antiarrhythmic therapy in geriatric patients is also unusual. Additionally, bradycardia has not been described among patients with Schizophrenia. Hence, we present a case of a patient who developed bradycardia and syncope following the commencement of low-dose donepezil in the inpatient psychiatric unit and subsequent resolution with cessation of donepezil.

## Case report

A 70-year-old Hispanic female presented to the psychiatric emergency room with worsening confusion, grossly disorganized behavior, forgetfulness, impaired activities of daily living, and inattentiveness. The patient’s family reported that her symptoms started a month prior to her presentation. Her outpatient provider also reported that during patient’s recent outpatient follow up visit, she demonstrated grossly disorganized behavior and reported noncompliance with her psychiatric medication regimen. The patient’s history was notable for schizophrenia, hypertension, hypothyroidism, dyslipidemia, and mild dementia.

On evaluation, the patient appeared disheveled with poor grooming. Her affect was constricted. She endorsed auditory hallucinations. Paranoid and persecutory delusions were elicited. Her speech was also markedly disorganized. Her chest radiograph, urine studies, and serum electrolytes were unremarkable. Head computed tomography (CT) at presentation revealed bitemporal lobe microvascular changes and electrocardiogram (EKG) was normal. She was recommenced on her home medications of haloperidol 5 mg orally twice per day and olanzapine 7.5 mg orally at bedtime. Psychosis improved with recommencement of her home medications in the inpatient psychiatric unit. However, she remained confused, and short and long-term memory loss persisted. Other patients reported that she was very distressed. She was evaluated by the neurology team and commenced a trial of donepezil 5 mg PO daily on the 7th day of inpatient psychiatric stay.

Baseline vital signs prior to the commencement of donepezil indicated blood pressure of 118/73 mmHg, pulse rate of 73 bpm, respiratory rate of 16 cpm, temperature of 98.4 F, and oxygen saturation of 98%. Basal metabolic rate (BMI) was 19.5 kg/m^2^. The baseline thyroid stimulating hormone (TSH) at the time of admission was normal at 2.69 mIU/L, and free thyroxine (T4) was 1.23 ng/dL.

On her 9th day of inpatient psychiatric admission, she was observed to have fallen in the day room but was immediately arousable. Her heart rate was 42 bpm, and her blood pressure was 110/76 mmHg. There was no head trauma. She was transferred to the telemetry unit.

The EKG done in the telemetry unit showed marked sinus bradycardia of 40 bpm. Her corrected QT interval (QTc) was unremarkable at 430 ms. Her blood pressure was normal in the telemetry unit. Chest radiograph showed no infiltrates or acute cardiopulmonary disease. Her hemoglobin (10.8 g/dL) and hematocrit level (33%) remained unchanged from baseline. The iron panel was normal except for a slight elevation in ferritin of 158 mg/L. The echocardiogram showed an ejection fraction of 60% with mild tricuspid and mitral valve regurgitation. Cardiac pacing or atropine was not indicated as her vitals remained stable. The carotid Doppler study showed no stenosis and repeat head CT imaging showed no acute changes. Fasting blood glucose was 118 mg/dL. All medications were stopped with an improvement of her heart rate to the 60 seconds over a 48 hour period. The patient did not have any repeat episodes of syncope or dizziness. Haloperidol 5 mg orally twice daily and olanzapine 7.5 mg orally at bedtime were recommenced on the 12th day of admission without any reduction in heart rate. Recommencement of donepezil on the 14th day reduced her heart rate from baseline 60s to late 40s and was identified as the causal agent.

She was subsequently discharged home from the medical unit on haloperidol 5 mg orally twice daily and olanzapine 7.5 mg orally at bedtime on the 20th day of admission. Patient followed up with outpatient cardiology and psychiatry clinic and recorded clinical improvement with stable vital signs.

## Discussion

Donepezil is a commonly used AI in inpatient and outpatient medical and neurological settings [6, 7]. There is also sparing use of donepezil in inpatient psychiatric settings to control mood and behavioral concerns. Though there is documented evidence of adverse gastrointestinal effects with donepezil pharmacotherapy [8], this was not evident in our patient. Instead, bradyarrhythmia was evident, which resolved following the termination of donepezil pharmacotherapy. Our patient did not have acute or chronic cardiovascular disease symptoms and was not on any cardiovascular medications with the potential for a drug-to-drug interaction that may manifest as bradycardia. Cases of drug-induced bradycardia attributable to donepezil therapy are rare in literature [9], especially among patients without history of antiarrhythmic medications.

We reviewed the literature on the EMBASE, PSYCHINFO, and PubMed databases regarding evidence on the adverse presentation of DIB. The search results showed a gap in the literature on the topic, especially regarding the psychiatric population without comorbid cardiovascular problems. Notably, evidence in the literature suggests synergistic pharmacodynamic effects of donepezil with other antiarrhythmic agents on heart rate [10]. Additionally, the literature’s findings also indicate that the role of genetics might be in play as inherited cardiac muscle defects have been reported as a risk factor [11]. Other reported risk factors are higher doses of antiarrhythmic, pharmacological naivety, and advanced in age [12]. On the basis of our case presentation, the only attributable factor present was the age of this patient. Considering that this was the initial exposure of donepezil for our patient, this also suggests that the notion of AI or psychotropic naivety may have been a probable cause of DIB. Hence, an incidental finding of bradycardia following 2 days of commencement of donepezil therapy, in the absence of any identifiable cause, is exceptional.

Bradycardia, a heart rate below 50–60 bpm, is potentially life-threatening, and its causality could be physiological or pathological in origin [8, 9, 12, 13]. Though different causes have been postulated in patients with comorbid cardiac problems and age-related differences, possible genetic predispositions have also been documented. There was no conduction disorder of the heart before the commencement of medications in our patient, which started following the initiation of a low dose of donepezil. Hence, we concluded that donepezil induced the observed changes. Additionally, geriatric patients with bradycardia may present with syncopal episodes or dizziness. Similarly, there are reports of increased falls and head trauma owing to comorbidities, gait impairment, and musculoskeletal deconditioning [14]. However, there is not enough literature with regards to this incident.

Regarding pathophysiology, the documented evidence suggests that dizziness, syncope, and bradycardia are more familiar adverse effects with rivastigmine [15] (another AI class). However, donepezil may cause bradyarrhythmia by increasing acetylcholine availability peripherally with cardiac muscarinic M2 receptor activation [16]. Considering that bradycardia is a rarely reported adverse effect of donepezil, AIs can exert their pharmacodynamic properties via vagotonic effects on the sinoatrial (SA) node, resulting in bradyarrhythmia [16]. Similarly, elderly patients on antiarrhythmics, especially beta-blockers and calcium channel blockers with augmented treatment using AI, are at greater risk for bradycardia [10]. Regarding the severity of the patient’s symptoms, medication dose tapering might be considered in mild bradycardia as the overall benefit may outweigh the adverse effect. In this case report, considering the acuteness of the presentation in a patient without any comorbid cardiovascular concern, we halted the medication with a successful resolution. Other authors have reported successful resolution following a switch to another AI, such as rivastigmine [17].

Our case provides evidence of the dearth of literature on bradycardia resolved after the halt of medications. Thus, this case is also unique because the patient was not on any cardiovascular medication with the potential for drug-to-drug interaction nor the capability of reducing the heart rate. This patient did not have any baseline cardiac abnormality before admission and commencement of medication, as evidenced by the typical EKG report, but was elderly. Notably, age had been described as a risk factor for DIB, but there is little evidence in literature to support it. Our finding is bolstered by a study by Bordier *et al*. [13], which reported that, among patients receiving donepezil therapy, 69% of those with syncope had an identifiable organic etiology. Hence, the dearth of literature on this occurrence.

## Conclusion

DIB is unusual in patients without background comorbid cardiovascular problems, and its incidence rate is unknown. Presently, there are no evidence-based alternative AI recommendations for incidental DIB. For the case presented here, we resolved DIB by stopping the medication and reappearance of symptoms following retrial confirmed causality. Hence, switching to oral or even a transdermal rivastigmine patch could be an alternate treatment plan for such a scenario. Alternatively, *N*-methyl-d-aspartate (NMDA) receptor antagonists, such as memantine, are also a safe alternative in patients with similar bradycardia events.

Presently, there is no baseline cardiac monitoring requirement guideline for patients on donepezil treatment. Hence, we infer that pre-assessment EKG should be considered before the commencement of AIs. Moreover, routine monitoring of a patient’s vital signs for at least the first 72 hours following the start of medication, irrespective of the baseline EKG findings, might be good practice for all psychiatrists. Extending this practice to inpatient and outpatient service settings will be worthwhile.

## Data Availability

Not applicable.
